# Comparative study of pavement anomaly detection using detection models with rotated bounding boxes

**DOI:** 10.1371/journal.pone.0329844

**Published:** 2025-08-12

**Authors:** Shunli Ji, Fusheng Niu, Yazhou Qin

**Affiliations:** 1 Department of Ship and Ocean Engineering, Jiangsu Shipping College, Nantong, China; 2 China Railway Tunnel Group Co. Ltd., Guangzhou, China; 3 School of Transportation and Civil Engineering, Nantong University, Nantong, China; Shandong University of Technology, CHINA

## Abstract

A comparative study on automated pavement anomaly detection is conducted to improve the detection accuracy of models based on You Only Look Once version 4-Tiny (YOLOv4-Tiny). This study is the first to introduce a rotated rectangle labeling strategy for pavement anomaly detection. The pavement image dataset, primarily collected in Nantong, China, includes 1,107 cracks and 691 potholes. First, the YOLOv4-Tiny model is trained and validated as a baseline, achieving a mean average precision (mAP) below 0.4, which is inadequate for practical use. To improve performance, the YOLOv4-ResNet50 model is proposed by replacing the original backbone with a ResNet50 network, resulting in modest gains. To further enhance precision, rotated rectangles—bounding boxes that include an additional rotation angle—are used instead of conventional axis-aligned boxes. Accordingly, the YOLOv4-Tiny-rotated and YOLOv4-ResNet50-rotated models are developed and evaluated on the same dataset. Results show that the YOLOv4-ResNet50-rotated model achieves an mAP of 0.742, outperforming the more advanced YOLOX model, which reaches an mAP of 0.627. Moreover, the rotated bounding boxes enable more accurate representation of the shape and orientation of inclined cracks, making this model particularly well-suited for pavement anomaly detection. This study demonstrates the effectiveness of rotated rectangle labeling and rotated predicted bounding boxes in detecting inclined cracks and potholes, laying a foundation for further research in this area.

## 1. Introduction

Road functionality degrades over time due to factors such as aging, overloading, and harsh weather conditions, leading to the formation of anomalies that can cause traffic accidents. Therefore, pavement anomaly detection is a crucial component of road management, operation, and maintenance to ensure driving safety and support logistics transportation. Traditionally, pavement anomaly data have been collected and categorized through manual inspection, which is time-consuming and inefficient, especially when dealing with low-resolution images. With advances in convolutional neural networks (CNNs) and computer vision techniques, autonomous object detection has been increasingly developed, alleviating the burden of real-time pavement anomaly detection [1–5]. These models can classify and localize anomalies promptly without the need to store large volumes of road image data, enabling remedial actions in time.

Generally, two types of object detection techniques are applied in the field of road engineering. The first type is two-stage object detectors, such as Region-Based CNN (R-CNN) [6] and its variants, Fast-RCNN [7] and Faster-RCNN [8]. These models process input image data in two stages: first, region proposals are generated (e.g., using selective search), and then these proposals are refined and classified by the detection model. The second type is one-stage object detectors, which simplify the procedure by processing the input image data in a single pass through the network. Examples include the You Only Look Once (YOLO) series [9–13] and Single-Shot MultiBox Detector (SSD) [14]. While two-stage detectors tend to achieve higher accuracy, one-stage detectors offer a better trade-off between inference speed and precision, making them more suitable for real-time applications and industrial deployment.

Excellent work has been done to establish benchmark datasets for pavement anomaly detection, particularly by Arya et al. [15–17]. The Road Damage Dataset 2022 (RDD2022), an extension of RDD2020, was developed to support benchmarking across different detection algorithms. RDD2022 includes pavement anomaly images collected from six countries—such as the US, China, Japan, and India—and comprises 47,420 images and over 55,000 annotated instances of pavement damage. This dataset enables low-cost, automated training, validation, and testing of pavement anomaly detection algorithms. In addition, the Crowd-Sensing-Based Road Damage Detection Challenge 2022 (CRDDC’2022) [18,19], held as part of the IEEE Conference on Big Data, provides a valuable platform to advance research and development in road damage detection techniques.

This study focuses on the implementation of the YOLO detector for pavement anomaly detection, taking advantage of its real-time capabilities. Li and Li [20] optimized the convolutional layers and spatial pyramid pooling (SPP) network in YOLOv8 and incorporated block attention to improve detection precision. Their improved YOLO detector achieved a mean average precision (mAP) of 0.817 for road defect detection. Tang et al. [21] proposed a lightweight YOLO-based model, named real-time pavement detection using YOLO (RPD-YOLO), which effectively reduces computational overhead while maintaining high speed and accuracy. Zhu et al. [22] evaluated six versions of the YOLO series on ground-penetrating radar (GPR) data and identified YOLOv5-s as the best model for detecting road defects such as voids and layer separations. A similar study on GPR images for road damage detection was conducted by Zhang et al. [23]. Ding et al. [24] proposed a custom YOLOv8-based model named shift-wise cross-scale dynamic head YOLO (SCD-YOLO) for pavement crack detection. Experimental results showed that SCD-YOLO improved the mAP by 4.1% compared to the baseline.

Although previous studies have laid a strong foundation for research in pavement anomaly detection, an important aspect has been largely overlooked concerning the rotated bounding boxes. Most existing work focuses on modifying detection model architectures or applying image data augmentation strategies to improve models’ performance. However, the impact of labeling strategies, especially for inclined cracks, has received little attention. Two factors contribute to the dearth of research in this area. The first one is that the rotated bounding box is not a built-in component in most existing detectors, and the second one is that it is more complicated to label with rotated rectangles than with axis-aligned labeling. Due to the characteristics of pavement anomalies—such as the commonly encountered inclined, narrow, and elongated cracks—a model with rotated bounding boxes is important for accurately capturing the orientation and shape of these defects. Although models with rotated bounding boxes have been successfully applied in other domains, it is worthwhile to evaluate their effectiveness in detecting pavement anomalies, given the unique characteristics of such defects.

The innovative aspects of this study can be summarized in two key contributions:

First, we replace the conventional axis-aligned rectangles with rotated rectangles to label inclined cracks and potholes. Correspondingly, we modify the baseline YOLO model to support training with rotated rectangle labels. Unlike models trained with axis-aligned labels—which output bounding boxes with four parameters—the modified model includes an additional parameter to represent the rotation angle of the bounding box. Our results demonstrate that rotated rectangle labeling is more effective, and the modified detection model significantly improves the mean average precision (mAP) for pavement anomaly detection.

Second, we evaluate four versions of detection models based on YOLOv4-Tiny. Although YOLOv4 is not the latest detection model, it remains widely used in edge devices in China due to its excellent tradeoff between inference speed and accuracy. Besides, several literatures revealed that the latest detection models are not the most suitable ones for pavement anomaly detection. For example, Kulkarni et al. [25] tested nine YOLO models, including YOLOv5, YOLOv6, and YOLOv7, on road images containing potholes, cracks, and humps. Their results showed that the YOLOv6-s model outperformed the others, achieving an mAP of 71.2% for road anomaly detection. Recently, Pham et al. [26] conducted a comparative study from YOLOv7 to YOLOv10 for road damage detection. Their results indicated that an ensemble of custom YOLOv7 models achieved the highest F1-score of 0.703 among all tested models. We use YOLOv4-Tiny as the baseline model, and then replace its backbone with a ResNet50 network, resulting in the YOLOv4-ResNet50 model. Both of these models are further enhanced using the rotated rectangle labeling strategy, leading to two improved models: YOLOv4-Tiny-rotated and YOLOv4-ResNet50-rotated. Finally, we compare our proposed YOLOv4-ResNet50-rotated model with a more advanced model, YOLOX, and show that our model outperforms YOLOX not only in precision but also in accurately capturing the shape and orientation of inclined cracks.

**Fig 1:**
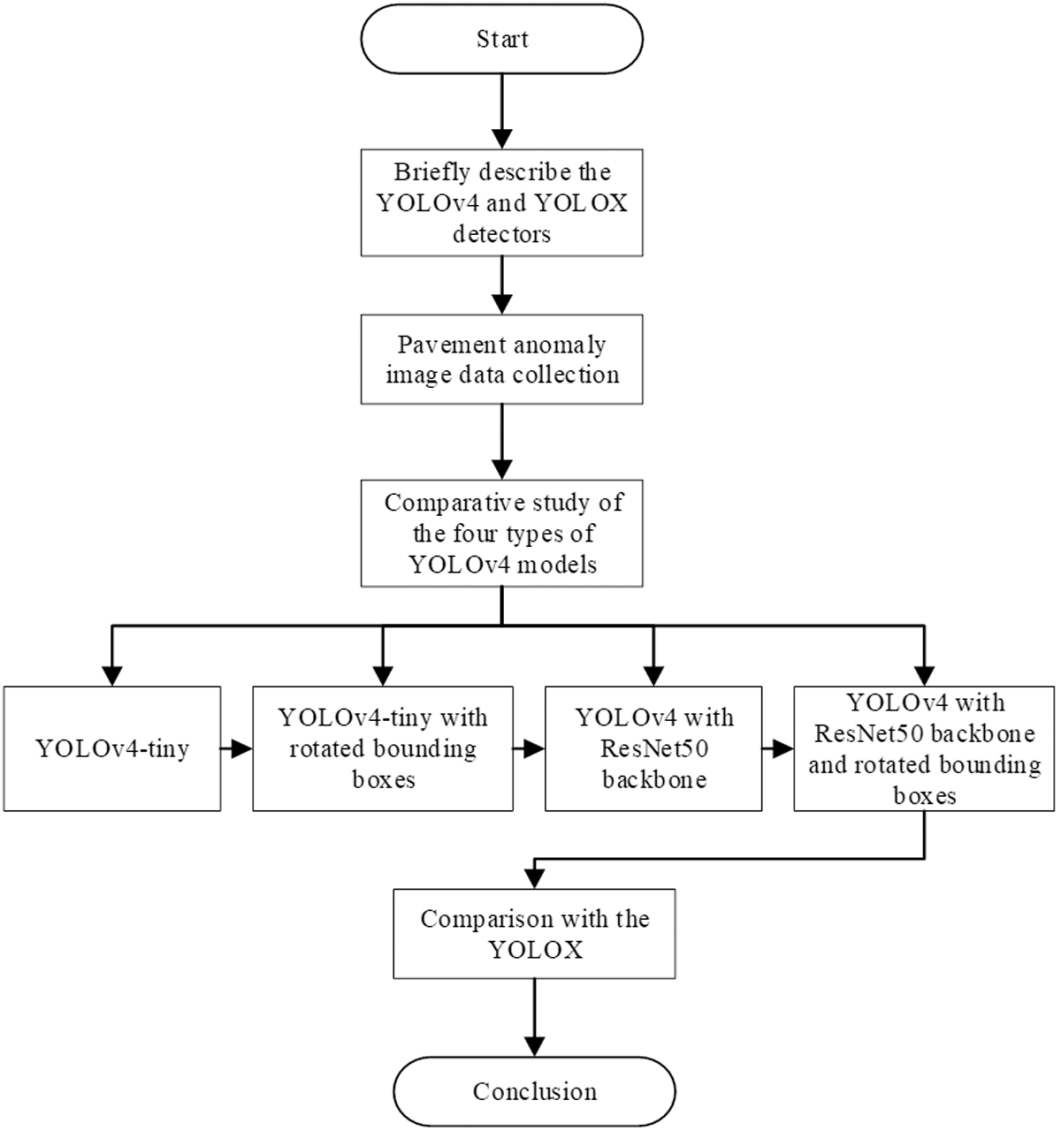
Flowchart of the study.

The overall workflow of this study is illustrated in Fig 1.

The paper is structured as follows. Section 2 introduces the methodology, and Section 3 describes the comparative analysis of the results of the four models, from training and validation to testing in detail. Section 4 compared the performance of our proposed model with the YOLOX and finally drew a conclusion in Section 5.

## 2. Methodology

**Fig 2:**
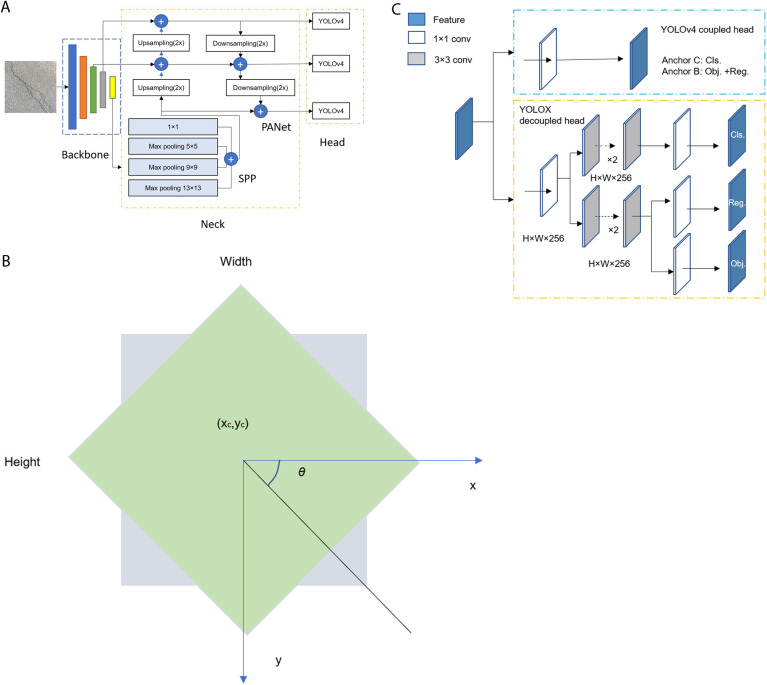
Diagram of the architecture of the YOLO detector. (a). YOLOv4 architecture. (b). rotated rectangle. (c). coupled and decoupled heads of the detector.

In this study, we use YOLOv4 as the baseline model, as it is widely deployed on edge devices due to its excellent balance between inference speed and precision. Fig 2a shows the architecture of YOLOv4, which includes a backbone, a neck, and three detection heads [12,27]. The original backbone of YOLOv4-Tiny is a compressed version of the Cross-Stage Partial (CSP) Darknet53, referred to as Darknet53-Tiny. It consists of 29 pretrained convolutional layers with 3 × 3 kernels. The backbone is responsible for extracting feature maps from the input image. Compared to its predecessors, YOLOv4 introduces key improvements, including a modified Path Aggregation Network (PANet) and Spatial Pyramid Pooling (SPP) blocks in the neck, as well as the use of bag of freebies and bag of specials techniques. Unlike the traditional PANet, which adds features from neighboring layers, the modified PANet applies concatenation instead. The SPP block increases the receptive field without reducing inference speed. As for the detection heads, YOLOv4 uses three head layers, while YOLOv4-Tiny uses only two to reduce the number of anchor boxes.

**Fig 3:**
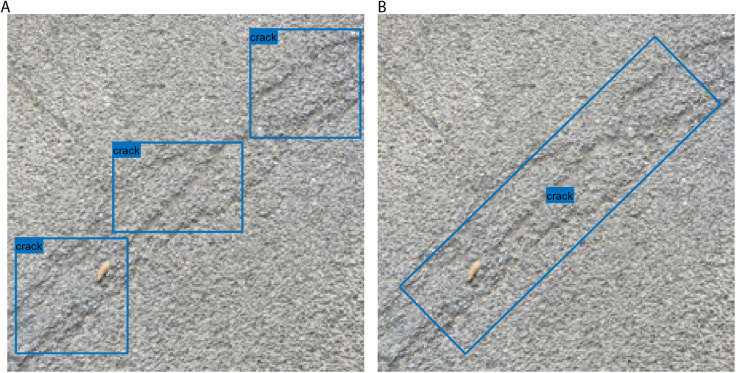
Two types of labeled boxes for inclined cracks: (a) axis-aligned rectangle; (b) rotated rectangle.

By using the rotated rectangle labeling technique, the predicted bounding box outputs of the modified detection models differ from those of the axis-aligned rectangle models, as shown in Fig 2b. While models with axis-aligned rectangles output four bounding box parameters in the format [x, y, w, h], representing the coordinates of the top-left corner, width, and height, the models with rotated rectangles output five parameters: the coordinates of the bounding box center, width, height, and the rotation angle. In Fig 2b, the gray bounding box represents the output from the axis-aligned rectangle model, while the green bounding box shows the output from the rotated rectangle model. The rotation angle *θ* is defined as shown in Fig 3b and ranges from 0º to 360º. Pavement anomaly images are imported into labeling tools, such as Image Labeler, where cracks and potholes are manually annotated using rotated rectangles. The labeled data are then exported as a separate file for training the detection models. The key optimizations required to enable the detection model to use rotated bounding boxes can be summarized in three aspects. First, the input layer is adjusted to accommodate the additional parameter, *θ*. Second, the output layer is modified to predict the corresponding rotation angle of the bounding box. Third, the loss function is updated to incorporate the angular loss between the predicted rotation angle and the ground truth.

Fig 2c shows the head architecture of YOLOX [13,27], an anchor-free, decoupled-head detection model, in contrast to YOLOv4-based models. The coupled head used in YOLOv4-Tiny, as shown at the top of Fig 2c, handles both regression and classification tasks using a single set of convolutional layers. It outputs the classification probability, objectness score, and bounding box coordinates. Since all three tasks share the same layers, this can lead to interference between them, potentially reducing overall performance. In contrast, the decoupled head used in YOLOX, shown at the bottom of Fig 2c, separates classification and regression into parallel branches, each with its own dedicated convolutional layers. This separation reduces task interference, leading to faster convergence and improved detection accuracy. Finally, this study compares the performance of the YOLOv4-ResNet50-rotated model with that of YOLOX to demonstrate that our proposed model with rotated bounding boxes achieves higher precision. It should be noted that the training time increased by approximately 10% for models using rotated rectangles—namely, the YOLOv4-Tiny-rotated and YOLOv4-ResNet50-rotated models—while the testing time on the test dataset remained nearly the same. The evaluation metrics used in this study include mAP at various intersection over union (IoU) thresholds, along with precision-recall curves. The metrics are defined as follows [28].


$$Precision=\frac{N_{TP}}{N_{TP}+N_{FP}}$$ (1)

$$Recall=\frac{N_{TP}}{N_{TP}+N_{FN}}$$ (2)

$$AP=\int_{0}^{1}P(R)dR$$ (3)

$$mAP=\frac{1}{n}\sum_{i=1}^{n}AP_{i}$$ (4)

Where *N*_TP_, *N*_FP_, and *N*_FN_ represent the number of true positives, false positives, and false negatives, respectively. *R* in Equation (3) represents recall, and *AP*_i_ denotes *AP* of the *i*th class.

## 3. Comparative study for four types of the detectors based on YOLOv4

In this study, several object detection models based on YOLOv4-Tiny are trained, validated, and tested. The baseline model, YOLOv4-Tiny, uses a lightweight backbone network pretrained on the Microsoft Common Objects in Context (MS COCO) dataset—a large-scale benchmark for object detection and segmentation, as described in [12,29]. To improve average precision (AP) for pavement anomaly detection, we replace the original backbone with ResNet50, naming the modified model YOLOv4-ResNet50. However, YOLOv4-ResNet50 still does not meet the required precision for real-world pavement detection. Furthermore, we found that using axis-aligned rectangles to label inclined pavement cracks—as illustrated in Fig 3—results in lower detection precision.

Fig 3 presents two labeling strategies for an inclined crack. In Fig 3a, the crack is labeled using a conventional axis-aligned rectangle, whereas Fig 3b shows the same crack labeled with a rotated rectangle. Clearly, labeling an inclined crack with a single rotated rectangle is more accurate than using multiple overlapping axis-aligned rectangles. Therefore, rotated rectangles are adopted to label two classes of pavement anomalies instead of conventional axis-aligned rectangles. Correspondingly, the improved YOLO detection models predict and localize the pavement anomalies with rotated bounding boxes. Based on this, we propose the YOLOv4-Tiny-rotated model, which is based on YOLOv4-Tiny but incorporates rotated bounding boxes, as well as the YOLOv4-ResNet50-rotated model, which extends YOLOv4-ResNet50 with the capability to detect inclined objects using oriented bounding boxes.

### 3.1. Data collection and augmentation

**Fig 4:**
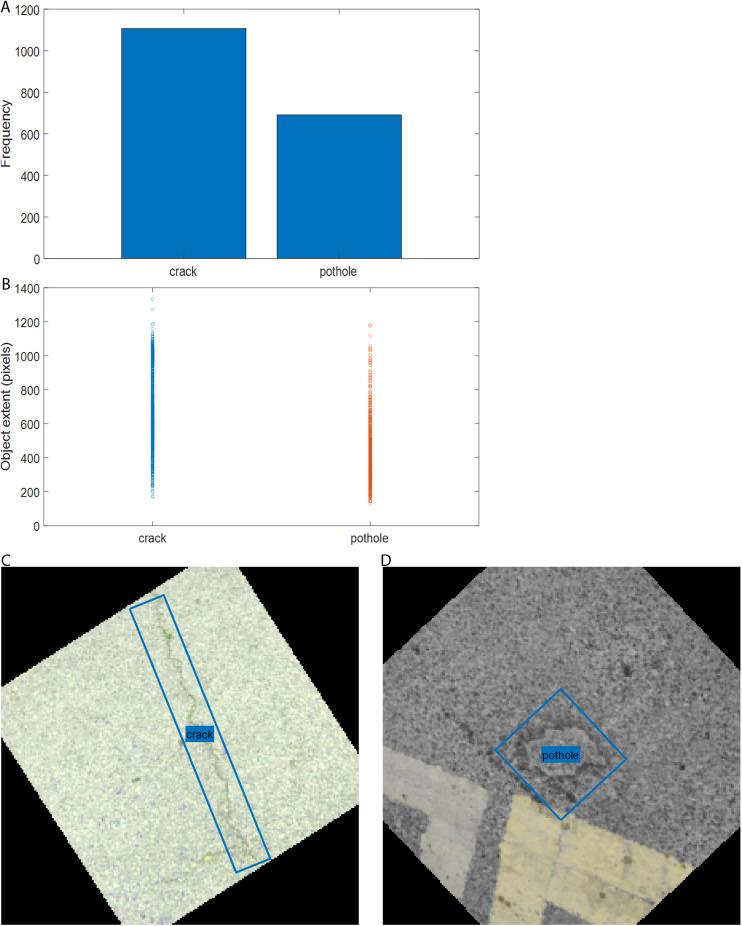
Overview of pavement anomaly data and image augmentation: (a) distribution of the two pavement anomaly classes; (b) distribution of anomaly sizes; (c) augmented crack image; (d) augmented pothole image.

Pavement anomaly image data were collected from several streets in Nantong, China. Upon inspection, it was observed that images containing potholes were significantly fewer than those containing cracks. To address this class imbalance, we supplemented the dataset with hundreds of pothole images downloaded from https://public.roboflow.com/object-detection/pothole. The final dataset consists of 727 images containing cracks (a total of 1,107 crack instances) and 442 images containing potholes (a total of 691 pothole instances), as shown in Fig 4a. To select appropriate anchor boxes for YOLOv4, it is necessary to assess the distribution of object sizes in the dataset before training the detector. The diagonal length of the labeled rectangles is used as the metric for evaluating object size. The distribution of object sizes is illustrated in Fig 4b.

Fig 4c, d show sample images after augmentation, performed prior to the training process. The augmentation techniques include horizontal flipping, translation along the horizontal and vertical directions, and rotation. These augmentations help improve the model’s robustness and reduce the risk of overfitting. Finally, the dataset is split into three parts in a 0.7: 0.15: 0.15 ratio for training, validation, and testing, respectively.

### 3.2. Training and model evaluation

**Fig 5:**
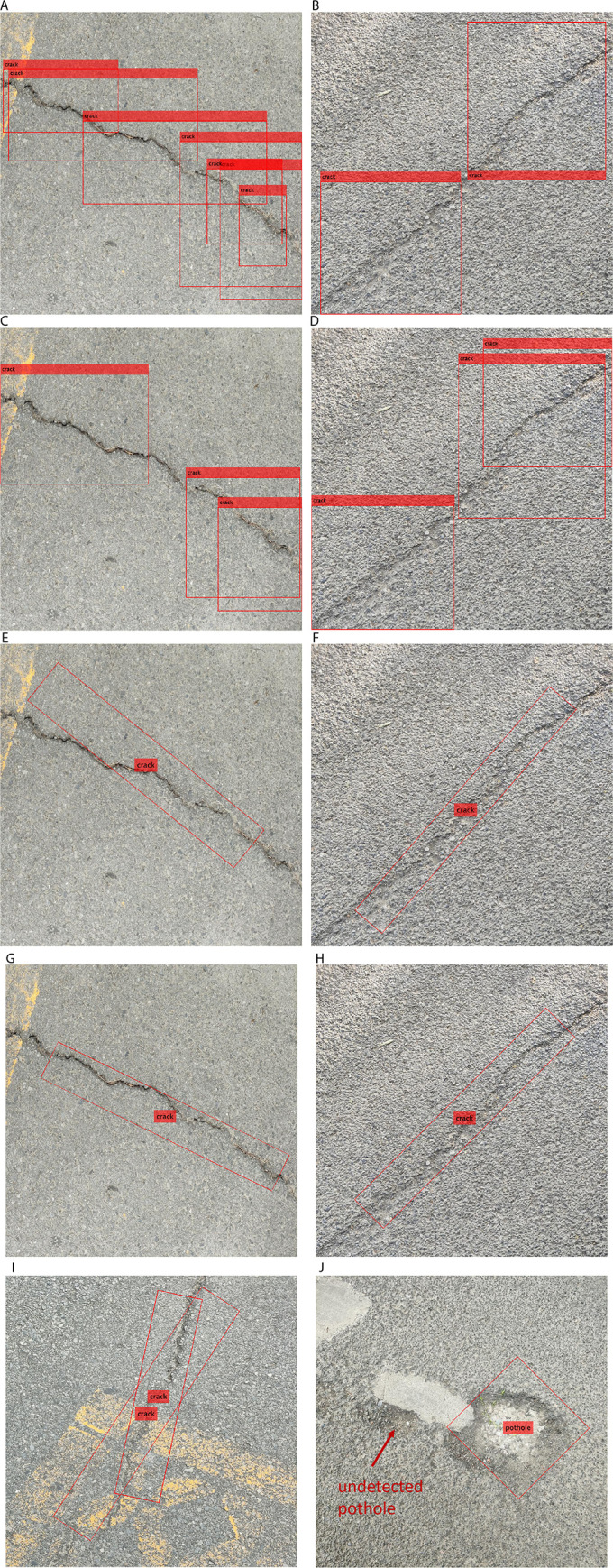
Continued.

The four object detection models are trained using nearly identical settings. The stochastic gradient descent with momentum (SGDM) algorithm is employed, with an initial learning rate of 0.0005. The mini-batch size is set to 8, the maximum number of epochs is 90, and the L2 regularization factor is 0.0005. Due to the anchor box-based nature of YOLOv4, the anchor boxes are estimated prior to training. Sample detection results from the four models are shown in Fig 5.

Fig 5a, b show two sample inclined cracks detected by the YOLOv4-Tiny model. It can be seen that the predicted bounding boxes overlap along the cracks, failing to accurately capture their location, shape, and orientation. The YOLOv4-ResNet50 model exhibits the same drawback, as shown in Fig 5c, d. Although there are fewer predicted bounding boxes in Fig 5c than in Fig 5a—indicating an improved detection ability—the model still cannot accurately represent inclined cracks using axis-aligned bounding boxes. In contrast, Fig 5e, f depict the same samples detected by the YOLOv4-Tiny-rotated model, and Fig 5g, h show results from the YOLOv4-ResNet50-rotated model. These four images demonstrate that models using rotated bounding boxes can precisely capture the shape and orientation of inclined cracks, making them more suitable for practical pavement anomaly detection. It should be noted that the YOLOv4-ResNet50-rotated model still has some drawbacks, which can lead to mismatches with the target pavement anomalies, as shown in Fig 5i, j. Specifically, Fig 5i shows a crack sample incorrectly detected with two rotated bounding boxes, while Fig 5j illustrates a case where only one of two potholes is correctly detected. These examples indicate that further improvements to the YOLOv4-ResNet50-rotated model are needed in the future.

**Fig 6:**
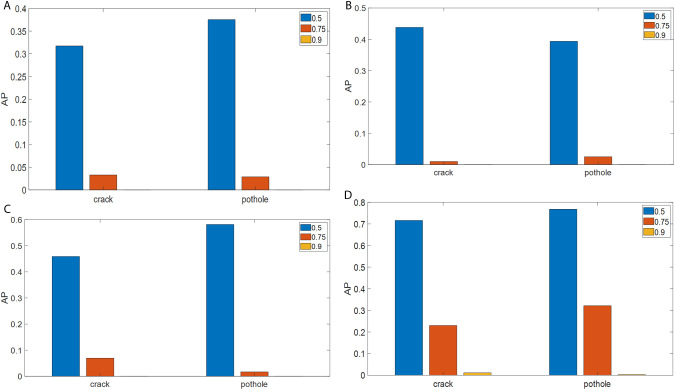
APs of different pavement anomaly detection models at three IoU thresholds: (a) YOLOv4-Tiny; (b) YOLOv4-ResNet50; (c) YOLOv4-Tiny-rotated; (d) YOLOv4-ResNet50-rotated.

Fig 6 presents the APs of the four models for the two types of pavement anomalies—cracks and potholes—at three IoU thresholds: 0.5, 0.75, and 0.9. As expected, higher IoU thresholds lead to lower AP values. Fig 6a shows the APs for cracks and potholes using the YOLOv4-Tiny model, all of which are below 0.4, indicating poor detection performance. Fig 6b shows that replacing the backbone with ResNet50 improves the AP_50_ by approximately 8%; however, the APs still remain below 0.5, suggesting that the YOLOv4-ResNet50 model is not sufficiently accurate for practical deployment.

Fig 6c shows the APs of the YOLOv4-Tiny-rotated model for crack and pothole detection. It can be observed that the AP_50_ values of the YOLOv4-Tiny-rotated model are significantly improved—by approximately 17% compared to the YOLOv4-Tiny model and by 10% compared to the YOLOv4-ResNet50. This demonstrates that the YOLOv4-Tiny-rotated model more accurately captures the shape and location of pavement anomalies using rotated bounding boxes. However, all AP values of the YOLOv4-Tiny-rotated model remain below 0.6, which is still insufficient for real-world pavement anomaly detection. Fig 6d presents the APs of the YOLOv4-ResNet50-rotated model. As shown, combining a stronger backbone with rotated rectangle labeling leads to significant improvements in detection performance. All AP_50_ values for both cracks and potholes exceed 0.7, with notable gains even at the 0.75 IoU threshold. These results suggest that the YOLOv4-ResNet50-rotated model meets the precision requirements for practical pavement anomaly detection.

**Fig 7:**
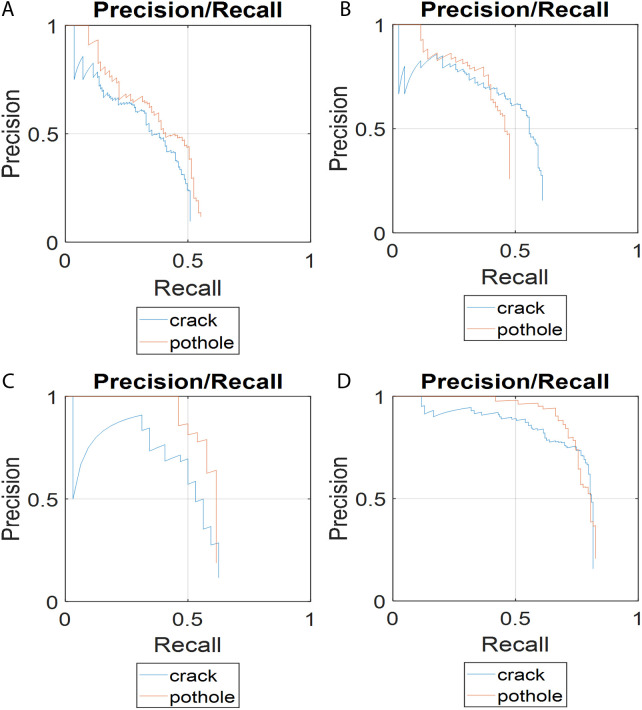
Precision-recall curves of different detection models: (a) YOLOv4-Tiny; (b) YOLOv4-ResNet50; (c) YOLOv4-Tiny-rotated; (d) YOLOv4-ResNet50-rotated.

Fig 7 shows the precision–recall curves of the four detection models for the two classes of pavement anomalies. Fig 7a illustrates the performance of the YOLOv4-Tiny model, which shows poor precision for both crack and pothole detection. Fig 7b presents the curves for the YOLOv4-ResNet50 model, which performs slightly better than the YOLOv4-Tiny but still does not meet the required precision levels. Fig 7c shows the curves for the YOLOv4-Tiny-rotated model, indicating improved performance over the previous two models. Finally, Fig 7d displays the results for the YOLOv4-ResNet50-rotated model, which significantly outperforms all other models in terms of precision.

Table 1 clearly presents the AP_50_, mAP, and other metrics of the four YOLO-based detection models for crack and pothole detection. The results indicate that the YOLOv4-ResNet50-rotated model outperforms the other models in detection precision and is suitable for real-world pavement anomaly detection.

**Table 1:** Performance of the four detection models.

	YOLOv4-Tiny	YOLOv4-ResNet50	YOLOv4-Tiny-rotated	YOLOv4-ResNet50-rotated
**AP**_**50**_ **for crack**	0.317	0.438	0.459	0.716
**AP**_**50**_ **for pothole**	0.375	0.394	0.582	0.768
**mAP**	0.346	0.416	0.520	0.742
**F1-score**	0.312	0.390	0.493	0.726
**Training time (Minute: Second)**	90:11	127:32	99:26	140:15

According to the detection results shown in Figs 5–7 and Table 1, the two detection models using rotated rectangle labeling and oriented bounding boxes performed better than the models using axis-aligned rectangles. As previously stated, rotated rectangles can more accurately label pavement anomalies, especially inclined cracks. As a result, the two models trained on data labeled with rotated rectangles can not only accurately locate inclined cracks but also capture their shape and orientation.

## 4. Compared with the YOLOX detection model

**Fig 8:**
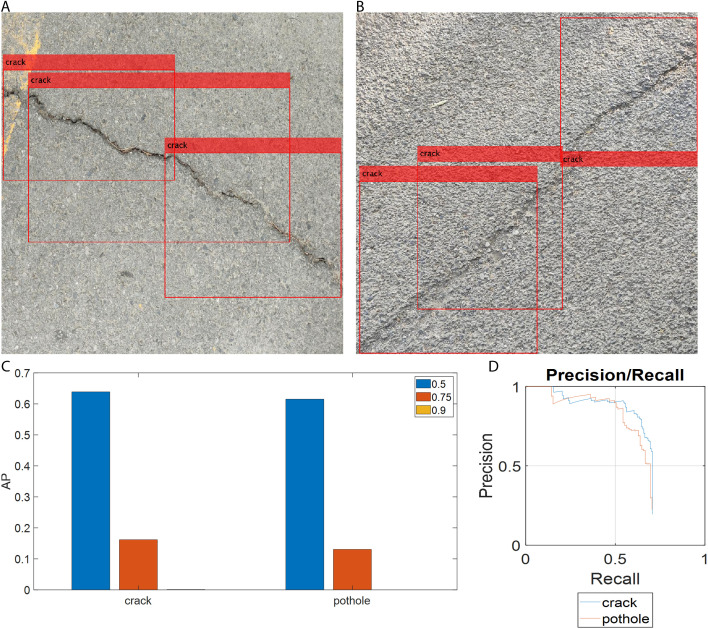
Detection results of YOLOX: (a) crack sample 1 detected by YOLOX; (b) crack sample 2 detected by YOLOX; (c) AP values of YOLOX for the two pavement anomaly classes; (d) precision-recall curve of YOLOX.

To further verify the efficacy of the YOLOv4-ResNet50-rotated detection model—which achieved the highest precision among the four models discussed in the previous section—we compared it with the more advanced YOLOX detection model. Since YOLOX is anchor-free, the pavement anomaly data labeled with rotated rectangles could not be directly used for training. Instead, we used the same axis-aligned rectangle-labeled image data that were used to train the YOLOv4-Tiny and YOLOv4-ResNet50 models. It is noted that the YOLOX model is trained by fine-tuning on the same dataset mentioned above. Fig 8a, b show detection results for samples 1 and 2 (the same samples shown in Fig 5) using the YOLOX model. As seen in these figures, the bounding boxes overlap along the inclined cracks, similar to the results of the YOLOv4-Tiny and YOLOv4-ResNet50 models. This indicates that, despite being a more advanced detection model, YOLOX also fails to capture the shape and orientation of inclined cracks. From this perspective, the YOLOv4-ResNet50-rotated model is superior to YOLOX.

**Fig 9:**
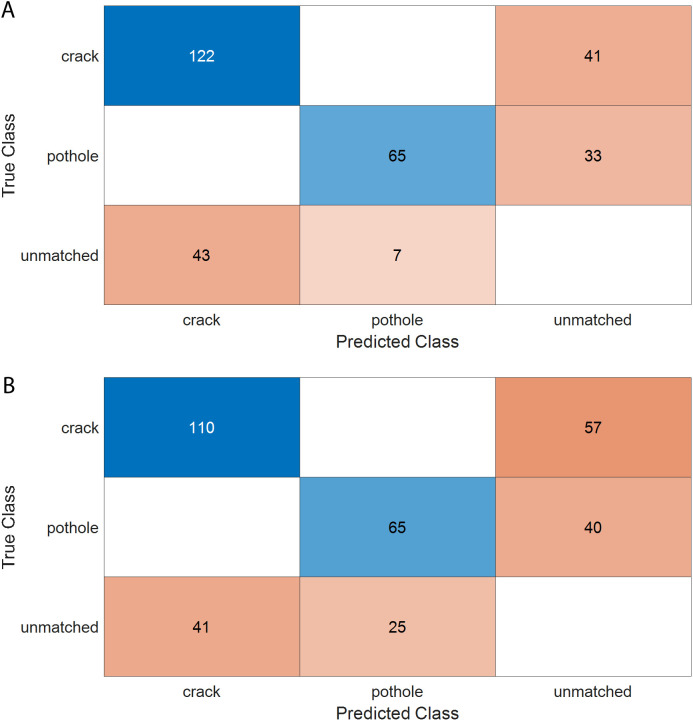
Confusion charts of YOLOv4-ResNet50-rotated and the YOLOX: (a) YOLOv4-ResNet50-rotated; (b) YOLOX.

Fig 8c shows the APs of the YOLOX model for crack and pothole detection at three IoU thresholds: 0.5, 0.75, and 0.9. As seen in Fig 8c, the AP_50_s of YOLOX are above 0.6, which is acceptable for practical pavement anomaly detection. However, compared with the YOLOv4-ResNet50-rotated model, the YOLOX model is inferior in detection precision. Fig 8d presents the precision-recall curves of the YOLOX model for both cracks and potholes. The AP_50_ for crack detection is close to that for pothole detection. The mAP of YOLOX at the IoU threshold of 0.5 is 0.627, which is lower than that of the YOLOv4-ResNet50-rotated model. In addition, the F1-score of YOLOX is 0.598, which is also lower than that of the YOLOv4-ResNet50-rotated model (0.726). Finally, a confusion matrix is used to compare the performance of the YOLOv4-ResNet50-rotated model and YOLOX, as shown in Fig 9.

Fig 9a presents the confusion matrix for the YOLOv4-ResNet50-rotated detection model. Unlike traditional classification confusion matrices, which compare predicted and actual classes, object detection confusion matrices also include an extra row and column for unmatched objects. These represent either ground truth objects that were missed (false negatives) or background regions mistakenly detected as objects (false positives). In Fig 9a, the model shows no confusion between cracks and potholes, as indicated by the absence of off-diagonal entries. However, the presence of unmatched objects lowers the model’s overall precision. Fig 9b shows the confusion matrix for the YOLOX model, which exhibits a similar pattern but with a higher number of unmatched objects, indicating comparatively lower detection performance.

By comparing the detection results of the YOLOv4-ResNet50-rotated model with YOLOX, it is evident that the YOLOv4-ResNet50-rotated model outperforms YOLOX—not only in terms of higher mAP scores but, more importantly, in its ability to capture the shape and orientation of inclined cracks. The results of this study illustrate that although the YOLOX model has a modern architecture, it is not the most suitable detector for pavement anomaly detection due to its lack of support for rotated bounding boxes. As a result, it cannot effectively capture the characteristics of pavement anomalies, especially inclined cracks and potholes. Furthermore, our findings are consistent with previous studies [25,26], which suggest that the latest object detection models are not necessarily the most effective for pavement anomaly detection tasks.

## 5. Conclusion

This study conducts a comparative evaluation of four YOLOv4-based detection models for pavement anomaly detection, using YOLOv4-Tiny as the baseline. The baseline model is then progressively improved to enhance its performance for practical application. First, by replacing the backbone with ResNet50, the baseline is transformed into the YOLOv4-ResNet50 model. Next, to improve detection precision, the axis-aligned bounding boxes are replaced with rotated rectangles in the YOLOv4-Tiny model, resulting in the YOLOv4-Tiny-rotated model. Finally, the two improvements are combined to form the YOLOv4-ResNet50-rotated model, which achieves an mAP of 0.742, significantly higher than the baseline model’s mAP of 0.346.

Predicted samples with rotated rectangles demonstrate that rotated bounding boxes are superior to axis-aligned ones, particularly for inclined cracks and potholes, as they more effectively capture the shape and orientation of the defects. The YOLOv4-ResNet50-rotated model is also compared with the more advanced YOLOX model. Results show that YOLOv4-ResNet50-rotated achieves a higher mAP than YOLOX in pavement anomaly detection and is suitable for deployment on resource-constrained edge devices. However, it should be noted that this study was conducted on a relatively small pavement image dataset collected in Nantong, China; therefore, the results should be generalized with caution. In future work, we plan to evaluate the proposed model on a larger pavement image dataset, such as RDD2022, while also accounting for environmental factors such as lighting variations, road markings, and plant shadows. Nonetheless, this study lays a foundation for further research in pavement anomaly detection, particularly in leveraging rotated bounding boxes to better capture the orientation and shape of surface defects.

## References

[1] Cui P, Bidzikrillah NA, Xu J, Qin Y. Application of the semi-supervised learning approach for pavement defect detection. Sensors (Basel). 2024;24(18):6130. doi: 10.3390/s24186130 39338875 PMC11435564

[2] Zuo C, Huang N, Yuan C, Li Y. Pavement-DETR: a high-precision real-time detection transformer for pavement defect detection. Sensors (Basel). 2025;25(8):2426. doi: 10.3390/s25082426 40285115 PMC12031190

[3] Jiang T-Y, Liu Z-Y, Zhang G-Z. YOLOv5s-road: road surface defect detection under engineering environments based on CNN-transformer and adaptively spatial feature fusion. Measurement. 2025;242:115990. doi: 10.1016/j.measurement.2024.115990

[4] Shin S-P, Kim K, Le THM. Feasibility of advanced reflective cracking prediction and detection for pavement management systems using machine learning and image detection. Buildings. 2024;14(6):1808. doi: 10.3390/buildings14061808

[5] Xu L, Fu K, Ma T, Tang F, Fan J. Automatic detection of urban pavement distress and dropped objects with a comprehensive dataset collected via smartphone. Buildings. 2024;14(6):1546. doi: 10.3390/buildings14061546

[6] Girshick R, Donahue J, Darrell T, Malik J. Rich feature hierarchies for accurate object detection and semantic segmentation. In: Proceedings of the IEEE Conference on Computer Vision and Pattern Recognition. 2014.

[7] Girshick R. editor Fast r-cnn. Proceedings of the IEEE international conference on computer vision. 2015.

[8] Ren S, He K, Girshick R, Sun J, Faster R. Towards real-time object detection with region proposal networks. Adv Neural Inform Process Syst. 2015;28. 10.1109/TPAMI.2016.2577031 27295650

[9] Redmon J, Divvala S, Girshick R, Farhadi A. You only look once: unified, real-time object detection. In: Proceedings of the IEEE Conference on Computer Vision and Pattern Recognition. 2016.

[10] Redmon J, Farhadi A. YOLO9000: better, faster, stronger. In: Proceedings of the IEEE Conference on Computer Vision and Pattern Recognition. 2017.

[11] Redmon J, Farhadi A. Yolov3: an incremental improvement. arXiv preprint. 2018. doi: 10.48550/arXiv.1804.02767

[12] Bochkovskiy A, Wang CY, Liao HYM. Yolov4: optimal speed and accuracy of object detection. arXiv preprint. 2020. doi: 10.48550/arXiv.2004.10934

[13] Ge Z, Liu S, Wang F, Li Z, Sun J. Yolox: exceeding yolo series in 2021. arXiv preprint. 2021. doi: 10.48550/arXiv.2107.08430

[14] Liu W, Anguelov D, Erhan D, Szegedy C, Reed S, Fu CY. Ssd: Single shot multibox detector. In: Computer Vision–ECCV 2016: 14th European Conference, Amsterdam, The Netherlands, October 11–14, 2016, Proceedings, Part I. Springer; 2016.

[15] Arya D, Maeda H, Ghosh SK, Toshniwal D, Sekimoto Y. RDD2020: an annotated image dataset for automatic road damage detection using deep learning. Data Brief. 2021;36:107133. doi: 10.1016/j.dib.2021.107133 34095382 PMC8166755

[16] Arya D, Maeda H, Ghosh SK, Toshniwal D, Sekimoto Y. RDD2022: a multi‐national image dataset for automatic road damage detection. Geosci Data J. 2024;11(4):846–62. 10.1016/j.dib.2021.107133 PMC8166755 34095382

[17] Arya D, Maeda H, Ghosh SK, Toshniwal D, Mraz A, Kashiyama T, et al. Deep learning-based road damage detection and classification for multiple countries. Automation Construct. 2021;132:103935. doi: 10.1016/j.autcon.2021.103935

[18] Arya D, Maeda H, Ghosh SK, Toshniwal D, Omata H, Kashiyama T. Crowdsensing-based road damage detection challenge (crddc’2022). In: 2022 IEEE international conference on big data (big data). 2022.

[19] Arya D, Maeda H, Sekimoto Y. From global challenges to local solutions: A review of cross-country collaborations and winning strategies in road damage detection. Adv Eng Inform. 2024;60:102388. doi: 10.1016/j.aei.2024.102388

[20] Li T, Li G. Road defect identification and location method based on an improved ML-YOLO algorithm. Sensors (Basel). 2024;24(21):6783. doi: 10.3390/s24216783 39517681 PMC11548543

[21] Tang H, Zhou D, Zhai H, Han Y. RPD-YOLO: a pavement defect dataset and real-time detection model. IEEE Access. 2024.

[22] Zhu J, Zhao D, Luo X. Evaluating the optimised YOLO-based defect detection method for subsurface diagnosis with ground penetrating radar. Road Mater Pavement Design. 2023;25(1):186–203. doi: 10.1080/14680629.2023.2199880

[23] Zhang Y, Lv H, Ni Y, Ye C, Wang D, Tang F. Automatic recognition of hidden road defects from GPR images using an enhanced CNN approach. J Transport Eng Part B. 2025;151(2):04025021.

[24] Ding K, Ding Z, Zhang Z, Yuan M, Ma G, Lv G. Scd-yolo: a novel object detection method for efficient road crack detection. Multimedia Syst. 2024;30(6). doi: 10.1007/s00530-024-01538-y

[25] Kulkarni S, Mittal N, Gupta RR, P RN. Investigation of YOLO models in the detection and classification of multiple negative road anomalies. In: 2023 14th International Conference on Computing Communication and Networking Technologies (ICCCNT). IEEE; 2023.

[26] Pham V, Ngoc LDT, Bui DL. Optimizing YOLO architectures for optimal road damage detection and classification: a comparative study from YOLOv7 to YOLOv10. In: 2024 IEEE International Conference on Big Data (BigData). IEEE; 2024.

[27] Terven J, Córdova-Esparza D-M, Romero-González J-A. A comprehensive review of YOLO architectures in computer vision: from YOLOv1 to YOLOv8 and YOLO-NAS. MAKE. 2023;5(4):1680–716. doi: 10.3390/make5040083

[28] Glučina M, Anđelić N, Lorencin I, Car Z. Detection and classification of printed circuit boards using YOLO algorithm. Electronics. 2023;12(3):667. doi: 10.3390/electronics12030667

[29] Wang CY, Bochkovskiy A, Liao HYM. Scaled-yolov4: Scaling cross stage partial network. In: Proceedings of the IEEE/CVF Conference on Computer Vision and Pattern Recognition. 2021.

